# Refinement of a colostrum-deprived pig model for infectious disease research

**DOI:** 10.1016/j.mex.2018.03.010

**Published:** 2018-04-10

**Authors:** Tanja Opriessnig, Priscilla F. Gerber, Patrick G. Halbur

**Affiliations:** a The Roslin Institute and The Royal (Dick) School of Veterinary Studies, University of Edinburgh, Midlothian, Scotland, UK; b Department of Veterinary Diagnostic and Production Animal Medicine, College of Veterinary Medicine, Iowa State University, Ames, IA, USA; c Animal Science, School of Environmental and Rural Science, University of New England, Armidale, New South Wales, Australia

**Keywords:** Colostrum-deprived pig model, Pig model, Infectious pathogens, Colostrum-deprived pigs, Naturally-farrowed, Artificially-reared

## Abstract

Well-defined pig models are useful to study the pathogenicity of newly recognized pathogens or strains in pigs and serve as animal models for some human diseases. The conventional pig model, where research pigs are sourced from commercial high-health production systems, is commonly used due to the easiness of getting pigs in a timely manner. However, freedom of the pig for the pathogen of interest is important at study start and serological assays to screen pigs for antibodies against newly identified pathogens or molecular assays detecting all possible circulating pathogen variants may not yet exist. Using colostrum-deprived (CD) pigs is a good alternative strategy to circumvent passively-acquired immunity against the pathogen of interest or exposure to pathogens shortly after birth. However, CD pigs are difficult to rear as they are highly susceptible to infections, and mortality rates in the first few days of life are often very high. Herein we report on refinement of a CD pig model with consistent survival rates of 90–100% of the piglets.

• Step-by-step protocol to derive and rear CD piglets with higher expected survival rates.
• Pig housing improvement minimizes the risk of disease transmission.
• Infectious virus disease research pig model purpose.

## Graphical abstract

**Figure:**
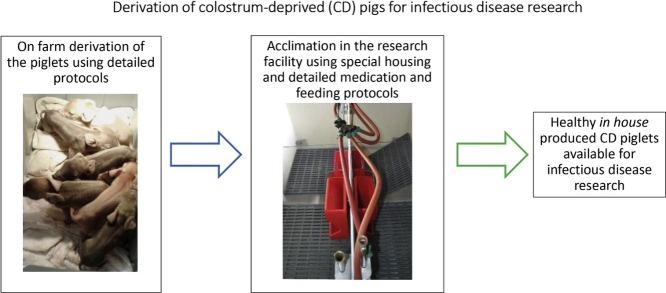

Specifications table

**Table:** 

Subject area	Veterinary science and veterinary medicine
More specific subject area	Laboratory animals
Method name	Colostrum-deprived pig model
Name and reference of original method	B. Ratcliffe, J.P. Fordham, A technique for rearing germfree piglets obtained without surgery. Lab. Anim. 21 (1987) 53–59.
	G.M. Allan, F. McNeilly, J.P. Cassidy, G.A.C, Reilly, B. Adair, W.A. Ellis, M.S. McNulty, Pathogenesis of porcine circovirus; experimental infections of colostrum deprived piglets and examination of pig foetal material. Vet. Microbiol. 44 (1995) 49–64.
	P.C. Gauger, K.M. Lager, A.L. Vincent, T. Opriessnig, M.E. Jr. Kehrli, A.K. Cheung, Postweaning multisystemic wasting syndrome produced in gnotobiotic pigs following exposure to various amounts of porcine circovirus type 2a or type 2b. Vet. Microbiol. 153 (2011) 229–239.
Resource availability	All consumables used are listed in Table 2, Table 3, Table 4. Specifications of the structures are described in the manuscript.

## Method details

**Fig. 1:**
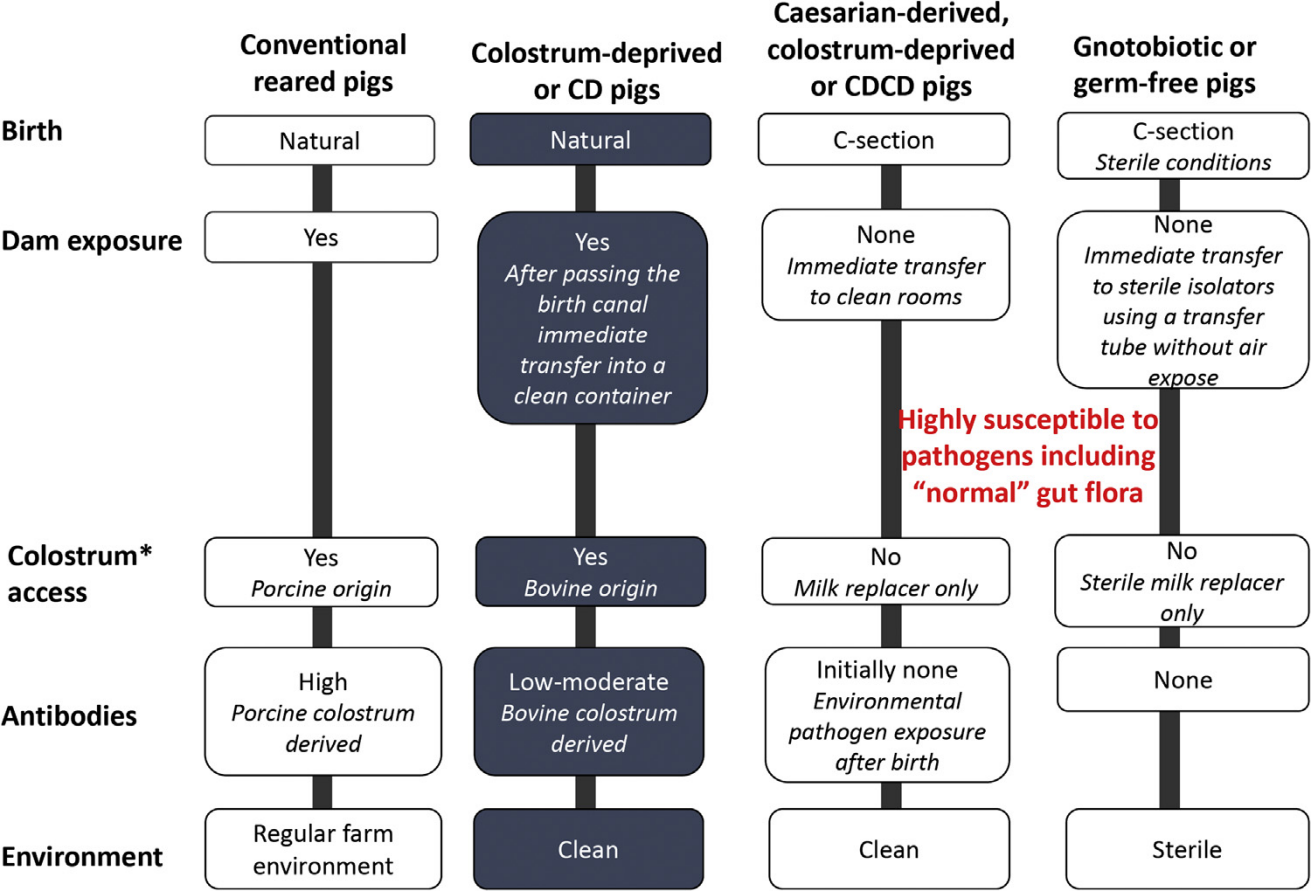
Comparison of the raising conditions of commonly used pig models used in infectious disease research. Colostrum is secreted from the time of farrowing until a few hours later and is rich in nutrients and immunoglobulins.

**Fig. 2:**
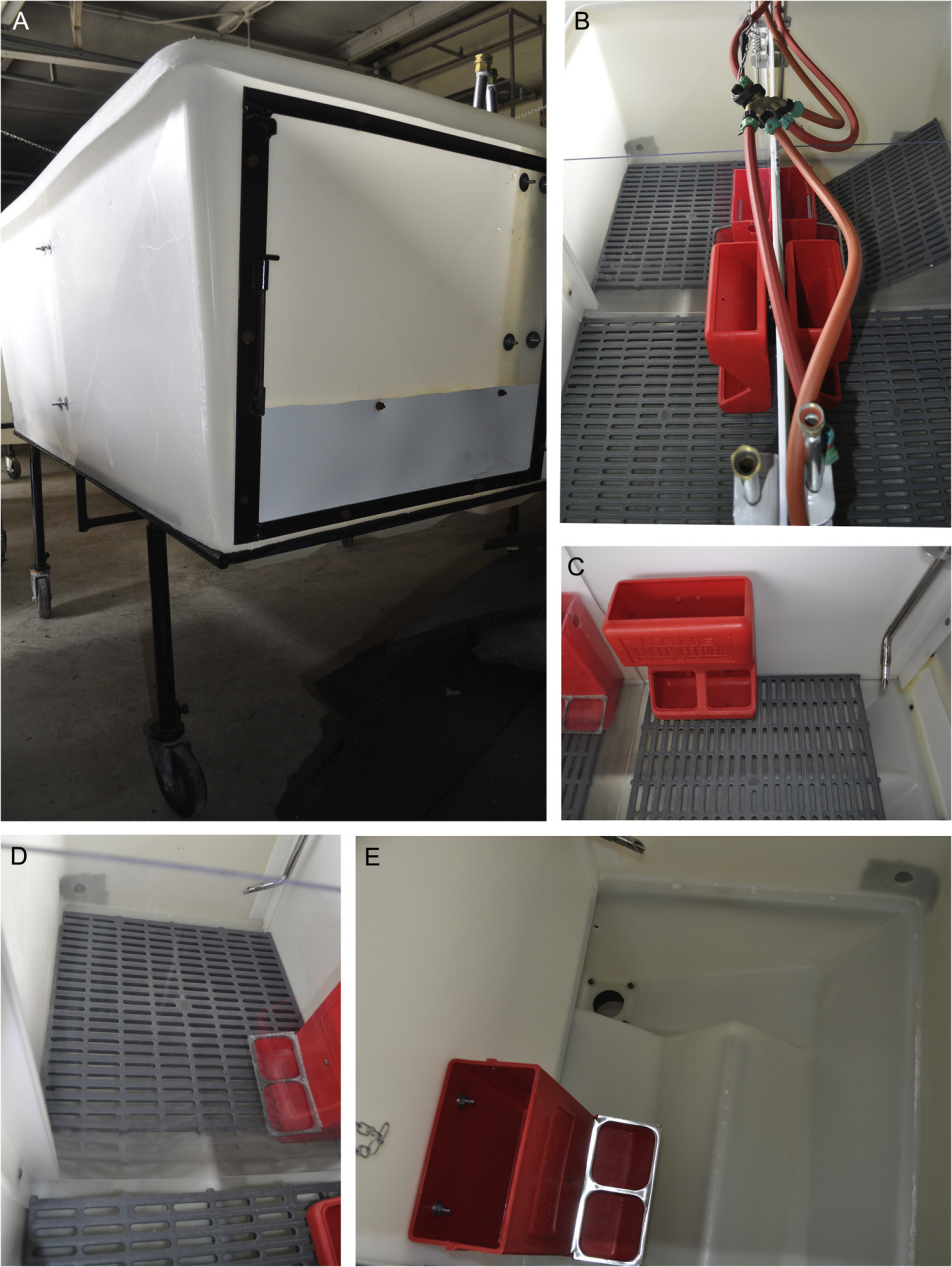
Housing of pigs in customized plastic tubs allows separate compartments for 1–2 pigs while allowing eye contact between pigs. **A.** Plastic tub from the front. The tub is raised from the floor. **B.** View into all four compartments of the plastic tub. **C.** Individual compartment with a self-feeder and a nipple drinker. **D.** Each compartment has a slatted plastic floor that can be easily cleaned and disinfected. **E.** Area underneath the slatted plastic floor demonstrating the drain system for waste removal.

Pig infectious disease researchers can chose to use conventional pigs, colostrum-deprived (CD) pigs [[1], [2], [3]], caesarian-derived colostrum deprived (CDCD pigs) or gnotobiotic/germfree pigs, each with their own advantages and disadvantages (Table 1; Fig. 1). Moderate to high mortality at an early age as the direct result of colostrum deprivation requires refinement of procedures [4] in sourcing and rearing pigs for research. The customized model for CD pigs described herein uses elevated pens split into quarters which can house 1–2 pigs per quarter (Fig. 2). This prevents direct contact between piglets and reduces movement of feces and waste between pens minimizing risk of transmission of diseases. The initial set-up of the research facility, on-farm derivation of piglets, preventive medical treatment protocols and a simplified feeding schedule, compared to previous protocols [1,5], are described in here. This refined model has been used at Iowa State University several times over the last few years resulting in survival rates of over 90% of the CD pigs derived for various research trials lasting from a few days to several weeks.

**Table 1:** Comparison of pig source, age at arrival, expenses and approximate waiting period for pigs when using different pig models currently available for infectious disease research.

^a^Pregnant sows are bought from a regular pig farm by the commercial research pig supplier.

^b^The litter size can range from 2–15 pigs and is unknown prior to surgery.

^c^Depends on available space including access to the surgery suite in the pig production company.

## Initial room set-up at the research facility

### Cleaning procedure

The rooms and equipment are washed and disinfected ahead of time using a degreaser followed by a disinfectant [6]. Rooms, walls and equipment are thoroughly cleaned with 70 °C water using a high-pressure (180 psi) nozzle and then sprayed with a degreaser (PRL-Grease Free; Pharmacal Research Laboratory Inc, Naugatuck, Connecticut), which is allowed at least 10–15 min contact with surfaces. Rooms and equipment are then fumigated with Virkon S (Dupont, Pharmacal Research Laboratories, Inc, Naugatuck, Connecticut) at the manufacturer’s recommended concentration and the room is allowed to completely dry (1–2 days).

### Room stocking

After the rooms are dry, it is stocked with personal protective equipment, supplies, and broad spectrum antibiotics, as described in Table 2, Table 3. Animal rooms are set up so that personnel enters through anterooms, where room specific boots, disposable N95 respirators, and disposable coveralls are stored.

**Table 2:** Supplies needed for different stages of the protocol.

	Type
**On-farm pig delivery**	• Latex gloves
	• Disinfecting wipes (Clorox®)
	• Towels (autoclaved cotton or paper towels)
	• Povidone scrub (VetOne®; 7.5%)
	• Plastic totes (1–2, large enough to hold several pigs; new)
	• Surgical scissors
	• Zip ties (VWR, Chicago, IL, USA)
	• Iodine spray (Triodine-7; Vedco)
	• Garbage bags (large)
	• Heat lamp and holder
	• Notepad and pen

**Arrival at the research facility**	• Colostrum replacer (Bovine IgG, Calf's Choice Total® Gold, SCCL, Saskatoon, SK, Canada)
	• Liquid milk replacer (1–2 cans per day and pig; Esbilac® Puppy Milk Replacer, PetAg, Hampshire, IL, USA)
	• Yogurt stored in a refrigerator
	• Pre-starter feed (Heartland All Natural Starter 2; Heartland Co-Op, West Des Moines, IA, USA)
	• Plastic spoons
	• Measuring cup (20–500 ml)
	• Rubber French catheter 8-gauge (Sovereign™, Tyco/Healthcare, Mansfield, MA, USA))
	• Catheter tip (60 ml)
	• Tube(s) for blood collection
	• Sufficient personal protective equipment (gloves, N95 respirators)
	• Needles (20 g by ¼, ½, or 1 in)
	• Syringes (1 ml, 3 ml, 5 ml, 12 ml, 20 ml)
	• Antimicrobials (described in Table 2)
	• Towels (autoclaved cotton or paper towels)
	• Feeding schedule copy
	• Eartags and eartagger (if more than a pig is housed in a compartment)

**Time of challenge**	• OB sleeves (Continental)
	• Needles (20 gauge by ½, ¾ or 1 in.)

**Table 3:** Medical supplies and treatment schedule.

Timing	Medication
At arrival, up to 6 h prior to challenge, and upon onset of clinical signs and repeated after 72 h if needed	Enrofloxacin (Baytril®100; 0.3 ml subcutaneously) and Ceftiofur crystalline free acid (Zoetis; 0.3 ml intramuscular)
At arrival and upon onset of clinical signs	*Clostridium perfringens* types C and D antitoxin (Colorado Serum Company, 3 ml orally and 3 ml subcutaneously, 25 ml of procaine penicillin are added to a 250 ml bottle)
Days 3 and 7	Injectable fortified iron (VetOne®; 100 mg intramuscular)

### Pig housing tubs

**Fig. 3:**
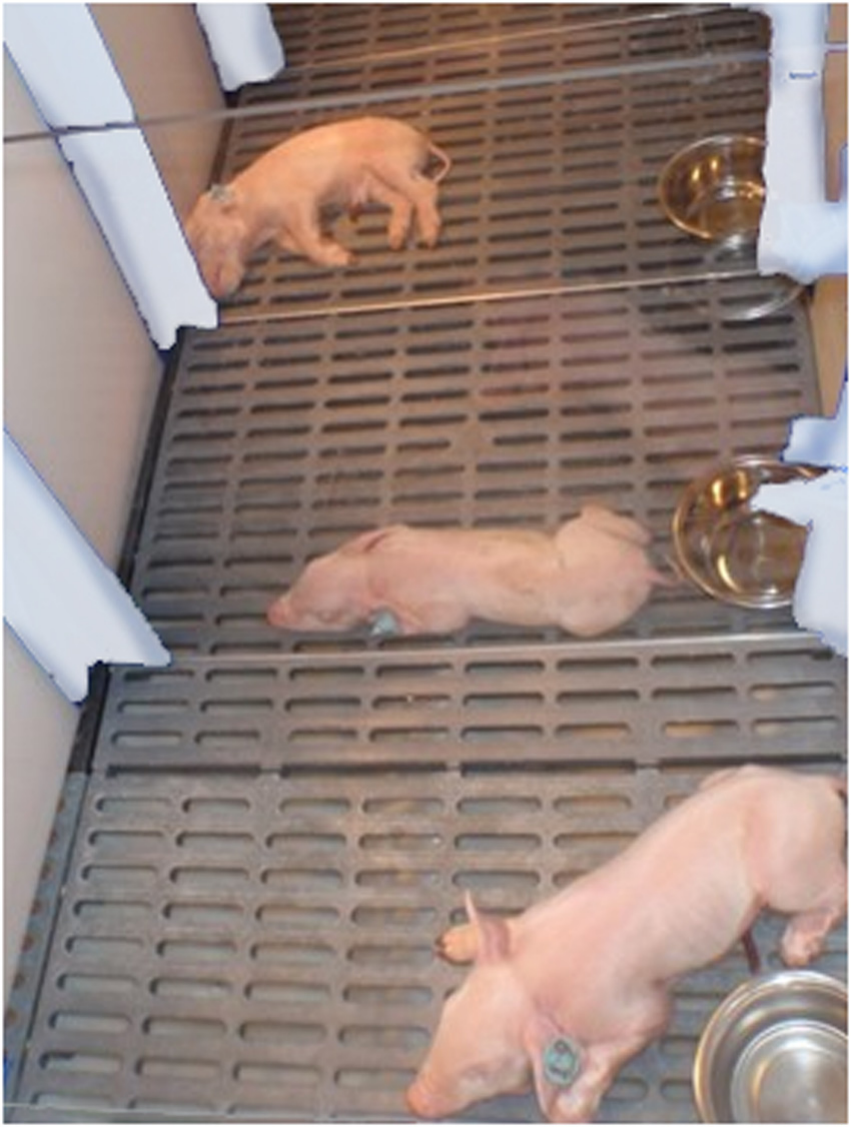
Piglets housed in the customized plastic tubs (image courtesy of Dr. Paulo Arruda).

In each animal room there is one tub made from heavy-duty, UV-stabilized polyethylene (Fig. 2) which is supported by a heavy-duty stand manufactured with a 22.2 mm solid rod steel, raising the unit 30.5 cm off the floor. The overall dimension of the tub is 91.5 cm (height) × 135 cm (width) × 170 cm (length). Below the floor is a 136 L capacity catch pan with a 7.6 cm slide gate for easy draining. Each tub is subdivided into four smaller compartments or pens equipped with clear plastic divisions which allow eye contact between pigs. Newborn pigs without teat access have a tendency to suckle on their littermates which can lead to inflammation and abscesses, often involving the umbilical cord stump. The presence of another pig, even if separated through a plexi-glass, may help to improve the welfare and comfort of the pigs (Fig. 3). For this reason, commonly only a single pig is housed in a compartment. However, it is possible to house up to 2–4 pigs per compartment which requires much closer monitoring of pigs for evidence of injuries and associated skin infections and less ability to accurately monitor milk and feed consumption per pig. Each compartment is equipped with a height adjustable heat lamp, a nipple drinker, a plastic feeder and environmental enrichment such as rubber or plastic dog toys. In addition, in the center of the tub (away from the heat lamp) is a sturdy ceramic cup for milk replacer. Rooms are heated and equipped well in advance of the arrival of the pigs.

### Temperature, humidity and photoperiod schedule

If possible, the environmental temperature and ventilation should be regulated by a ventilation system to approximately 30–40% to avoid skin infections. The temperature in the utilized rooms is set to 30 °C from day 1 to day 10 and is reduced by 1 °C per week thereafter. The heat lamp is turned off on day 28 or sooner based on pig behavior. The photoperiod is set to 12 h light and 12 h dark.

### Personal movement

To minimize biosecurity risks, any personnel that enter the animal room is required to shower and wear disinfected boots and clean coveralls to walk from the shower room to the animal room. Boots are removed prior to the entrance into the animal ante-room. Disposable coveralls, N95 respirators, and designated room-specific boots are put on in the ante-room prior to entrance into the animal rooms and disposable gloves are put on after entering the animal room.

## On-farm derivation of newborn piglets

### Farm selection

Depending on the requirement of the research being conducted, dams are sourced from high-health status farms that routinely monitor for common vertically transmitted viruses such as porcine reproductive and respiratory syndrome virus, porcine parvovirus and porcine circovirus type 2. Serum or other appropriate sample types from a representative number of sows is collected in advance for screening against the pathogens of interest.

### Selection and preparation of pregnant dams

**Fig. 4:**
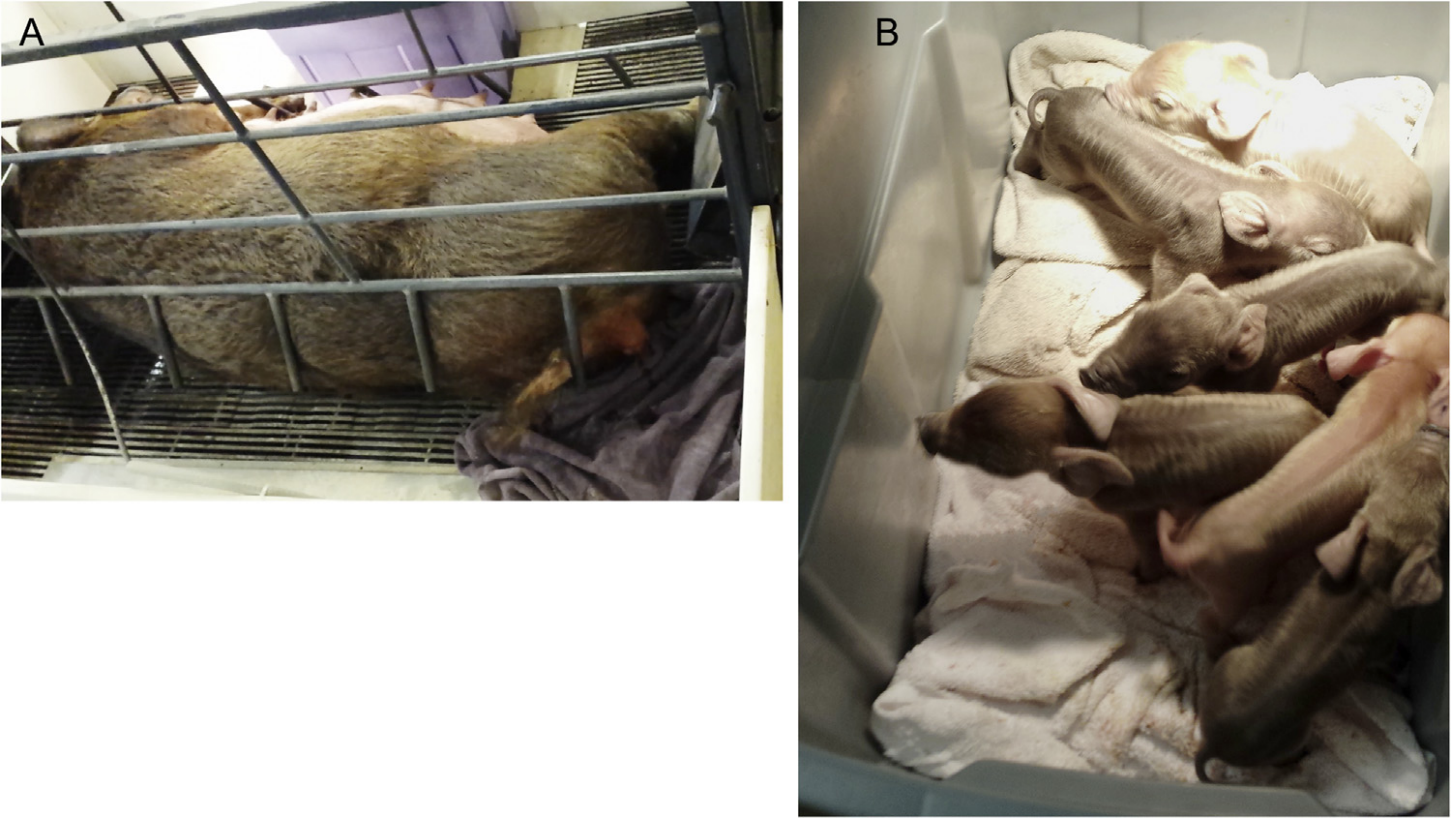
On-farm derivation of newborn piglets. **A.** Preparation of the dam with a view of the transport tote by the side of the crate. **B.** Farrowed piglets in the tote under the heating lamp.

Depending on the number of piglets needed, several pregnant dams close to farrowing (presence of colostrum in mammary glands; behavioral changes), housed individually in farrowing crates on a commercial sow farm geographically close to the research facility are pre-selected. For the selected dams, the vulva is cleaned with a povidone scrub and a clean cotton towel is placed below the vulva to decrease contamination (Fig. 4A). If the towel is contaminated by fecal material or saturated with fluid, it is replaced. The selected dams are closely monitored in order to avoid contact between the newly born piglets and the floor or dam. Having multiple people is advantageous.

### Piglet management during farrowing

As the piglets are coming out of the birth canal, they are picked up using clean gloves without touching the farrowing crate or the floor, dried off with a clean towel, their body is wiped with a povidone scrub, and they are put in a new large plastic tote with clean towels positioned under a heat lamp. Alternatively, rags slightly moistened with 70% ethanol may also be used to clean the pigs. The strongest and healthiest pigs are selected for the project. The tote is placed on a large plastic bag in a clean area of the barn away from the farrowing area (Fig. 4B). The umbilical cord of any pig bleeding excessively is tied with a clean zip tie. Once the required number of pigs has been collected, the tote with the pigs is transported immediately to the research facility.

## Arrival at the research facility

Upon arrival, a zip tie is placed on the umbilical cord, approximately 2.5 cm from the body wall, and the umbilical cord stump is sprayed with iodine. Each pig is treated with antimicrobials as specified in Table 3, receives a commercial colostrum replacer of bovine origin (Table 2, Table 4), and 1–2 pigs are placed in each compartment of the plastic tubs (Fig. 3). Using a bovine colostrum replacer will provide the piglets with antibodies against some common pathogens. On the day of arrival, appropriate sample types such as serum or feces can be collected from the piglets and tested to assure freedom of pathogens of interest prior to initiation of the experiment.

**Table 4:** Feeding schedule. Milk replacer feeding schedule adapted from protocols obtained through the National Animal Disease Center, USDA, Ames, Iowa [7]. Abbreviations used: C = Colostrum (including the amount given); M = Milk replacer (including the amount given); Y = One tablespoon of plain natural yogurt; F = One tablespoon of pre-starter feed.

Day	Morning	Noon	Evening	Total C and M per day
0	90 ml C are given initially and once completed this is followed by 10–30 ml M every 2–3 h			**270 ml**
1	10–30 ml M are given every 3–4 h			**270 ml**
2	140 ml M	80 ml M	140 ml M	**360 ml**
3	145 ml M, Y	70 ml M	145 ml M, Y	**360 ml**
4	150 ml M, Y	60 ml M	150 ml M, Y	**360 ml**
5	160 ml M, Y	40 ml M	160 ml M, Y	**360 ml**
6	165 ml M, Y	30 ml M	165 ml M, Y	**360 ml**
7	170 ml M, Y, F	20 ml M	170 ml M, Y, F	**360 ml**
8	200 ml M, Y, F	–	200 ml M, Y, F	**400 ml**
9	210 ml M, Y, F	–	210 ml M, Y, F	**420 ml**
10	220 ml M, Y, F	–	220 ml M, Y, F	**440 ml**
11	230 ml M, Y, F	–	230 ml M, Y, F	**460 ml**
12	240 ml M, Y, F	–	240 ml M, Y, F	**480 ml**
13	250 ml M, Y, F	–	250 ml M, Y, F	**500 ml**
14	250 ml M, Y, F	–	250 ml M, Y, F	**500 ml**
15	260 ml M, Y, F	–	260 ml M, Y, F	**520 ml**
16	270 ml M, Y, F	–	270 ml M, Y, F	**540 ml**
17	280 ml M, Y, F	–	280 ml M, Y, F	**560 ml**
18	290 ml M, Y, F	–	290 ml M, Y, F	**580 ml**
19	300 ml M, Y, F	–	300 ml M, Y, F	**600 ml**
20	310 ml M, Y, F	–	310 ml M, Y, F	**620 ml**
21	320 ml M, Y, F	–	320 ml M, Y, F	**640 ml**
22	330 ml M, Y, F	–	330 ml M, Y, F	**660 ml**
23	340 ml M, Y, F	–	340 ml M, Y, F	**680 ml**
24	350 ml M, Y, F	–	350 ml M, Y, F	**700 ml**
25	360 ml M, Y, F	–	360 ml M, Y, F	**720 ml**
26–42	370 ml M, Y, F	–	370 ml M, Y, F	**740 ml**

## Feeding schedule

### Colostrum

Colostrum replacer is only given once upon arrival of the pigs to the research facility. The colostrum replacer is prepared according to the instructions of the manufacturer using hot water (approximately 60 °C) and a clean plastic dish, and the piglets are syringe-fed when the mixture has cooled down to body temperature. The amount of colostrum fed depends on the product and the willingness of the pig to eat. Commercially available spray-dried bovine colostrum products are not sterile. A disposable syringe is filled with the colostrum replacer and the piglet is held carefully with the index finger at the angle of the jaw, forcing the mouth open while the palm of the hand is around the back of the head. The colostrum is squirted into the back of the piglet’s mouth at a rate that allows the pig to suckle and swallow the milk with a maximum amount of 25–30 ml colostrum or milk replacer during the first 2–3 days. Alternatively, piglets can be fed with disposable bottles. However, usage of syringes allows a more precise monitoring of the volume ingested by each pig. Alternating the piglets after 5–10 ml can be helpful when getting them started with syringe feeding. As a last resort, if syringe feeding is not possible, a rubber stomach feeding tube (Table 2) is used; however, this involves a higher risk of injury to the piglet and should be avoided if possible. If necessary, the feeding tube is slowly inserted over the back of the tongue, and the pig is observed carefully for a swallowing reflex before continuing with the procedure. If accidental introduction of the tube into the trachea occurs, the pig will start coughing and the tube needs to be immediately withdrawn. Once the tube is in the pig’s stomach a syringe is used to pump the food into the pig. A list in which feeding times and amount of food being consumed are tracked is helpful, especially during the first days of life.

### Milk replacer

After the first feeding, all subsequent feedings consist of commercially available sterile liquid milk replacer (Table 4) as described [7] providing a portion orally by a disposable syringe (20–40 ml) or a disposable bottle and the remainder in ceramic bowls. Syringe or bottle feeding is only done for the first 2–3 days at which time the pigs readily take milk from the bowl. Any milk leftover between feedings is discarded, and the bowl is washed with water and cleaned with a disinfectant wipe prior to re-fill. Milk disappearance and body condition are monitored closely. Any reduction in milk consumption could indicate that a pig may require additional care such as assisted feeding (syringe or tube feeding) or medication. If all milk is gone upon arrival for the next feeding, the amount of feed needs to be increased as appropriate (typically 10%) and recorded on the feeding sheet. The timing of the feedings is important; however, 1–2 h of variation between feedings is acceptable.

### Feed supplementation after 3 days of age

If piglets are being held longer than 3–5 days, one tablespoon of plain yogurt is added to the milk twice a day. If piglets are being held longer than 7–10 days one tablespoon of pelleted pre-starter feed is added to the milk at each feeding time to create a gruel.

### Personnel required

Feeding of the pigs and cleaning of the equipment and pens can be easily done by a single person. A shower and clothing change is required between rooms. During the first two days after the pigs arrive when individual feeding is required every few hours, having additional people is helpful.

## Pig maintenance during the trial period

### Medication schedule

The pigs receive intramuscular injections of iron at 3 and 7 days of age (Table 3) and they are prophylactically treated with antimicrobials 6 h ahead of viral challenge. Once the pigs have been inoculated, disposable OB sleeves (Table 2) that are changed between each pig are put on over the disposable coveralls and latex gloves. Clinical signs including inappetence, lethargy, diarrhea, labored respiration, fever, loss of body condition or dehydration can appear quickly and almost always require immediate intervention. In addition to antibiotics, dehydrated animals can be given balanced electrolyte solutions.

### Cleaning of the pig housing

The cleaning of the pens during the trials is typically limited to washing the ceramic bowls with hot water followed by cleaning them with disinfectant wipes. On rare occasions, visibly contaminated areas, including feeders, are wiped by hand using a disinfectant towel. The fecal material and urine produced by the pigs is collected underneath the raised tubs and removed at the termination of the trial.

### Additional information

Infectious disease research is often hindered by the presence of the pathogen of interest or due to presence of antibodies against that pathogen. Sows have an epitheliochorial placenta [8], which prevents the transfer of maternal antibodies to the offspring through the placenta and piglets are born free of antibodies. Antibody transfer from the dam to the piglet occurs via colostrum to protect pigs at a young age [9]. Colostrum supplies a pig with energy and protein and depending on the exposure and vaccination history of the dam, immunoglobulins to agents other than the pathogen of interest for the research project. This makes CD and CDCD pigs extremely susceptible to develop project-unrelated diseases that often cause high mortality [5] and model refinement is needed.

Sourcing and raising protocols for CD piglets should and can be adapted based on the purpose of the research. Protocols used for rearing of CD pigs as a newborn infant model for studying immune responses [10,11] or neurological development [12] have been recently described. Although several manuscripts report the use of CD pigs for study of infectious pathogens, few detail the rearing conditions [1,5,13,14]. Rearing protocols for CD piglets with limited or no use of antibiotics and more intensive feeding schedules have been previously described [1,5] and would be more appropriate for bacterial challenge studies. Raising and housing CD piglets under the conditions described in this manuscript typically results in high survival rates and these pigs can be used at an early age to study non-vertically transmitted pig viruses.

## Funding

Funding was received by the Biotechnology and Biological Sciences Research Council (BBSRC) Institute Strategic Programme Grant awarded to the Roslin Institute (BB/J004324/1; BBS/E/D/20241864).

## Conflict of interest

None declared.
